# Development of phenotypic assays for identifying novel blockers of L-type calcium channels in neurons

**DOI:** 10.1038/s41598-020-80692-5

**Published:** 2021-01-11

**Authors:** Rebecca Hagan, Elizabeth Rex, David Woody, Monika Milewski, Thomas Glaza, Michael P. Maher, Yi Liu

**Affiliations:** 1 Neuroscience Discovery, Janssen Research & Development, L.L.C, 3210 Merryfield Row, San Diego, CA 92121 USA; 2 Discovery Sciences, Janssen Research & Development, L.L.C, 3210 Merryfield Row, San Diego, CA 92121 USA

**Keywords:** Biological fluorescence, Phenotypic screening, Receptor pharmacology, Ion transport

## Abstract

L-type calcium channels (LTCCs) are highly expressed in the heart and brain and are critical for cardiac and neuronal functions. LTCC-blocking drugs have a long and successful record in the clinic for treating cardiovascular disorders. In contrast, establishment of their efficacy for indications of the central nervous system remains challenging given the tendency of existing LTCC drugs being functionally and mechanistically more selective for peripheral tissues. LTCCs in vivo are large macromolecular complexes consisting of a pore-forming subunit and other modulatory proteins, some of which may be neuro-specific and potentially harbor mechanisms for neuronal selectivity. To exploit the possibility of identifying mechanistically novel and/or neuro-selective blockers, we developed two phenotypic assays—a calcium flux-based primary screening assay and a patch clamp secondary assay, using rat primary cortical cultures. We screened a library comprised of 1278 known bioactive agents and successfully identified a majority of the potent LTCC-blocking drugs in the library. Significantly, we identified a previously unrecognized LTCC blocker with a novel mechanism, which was corroborated by patch clamp and binding studies. As such, these phenotypic assays are robust and represent an important step towards identifying mechanistically novel and neuro-selective LTCC blockers.

## Introduction

L-type calcium channels (LTCCs, a.k.a. Ca_V_1) belong to a family of voltage-gated calcium channels (VGCCs), which selectively permit Ca^2+^ ions into cells upon opening in response to membrane depolarization. LTCCs (predominantly Ca_V_1.2 and Ca_V_1.3) are widely expressed and serve critical functions in both brain and heart/vasculature^1^. Gain-of-function mutations in Ca_V_1.2 and Ca_V_1.3, for example, can lead to Timothy syndrome^2^ (with multiorgan dysfunction, including prolonged QT, cardiac arrhythmias and autism) and neurodevelopmental or endocrine symptoms^3^. Ca_V_1.2 and Ca_V_1.3 are also implicated in psychiatric and neurological disorders such as bipolar disorder, major depression and Parkinson's disease^4,5^.

Drugs that block LTCCs for the treatment of cardiovascular disorders such as hypertension and arrhythmia have seen successful clinical use for decades^6^. In contrast, establishment of the clinical efficacy of LTCC-blocking drugs remains challenging for disorders of the central nervous system (CNS), such as bipolar disorder^7^. One hypothesis holds that such drugs (even those that are highly CNS penetrant) may not reach therapeutic concentrations in the brain at therapeutic dosages for treating cardiovascular diseases^6^. Although all known LTCC drugs bind to the pore region of α1 subunits, functional potency in vivo appears to vary substantially in the brain and cardiovasculature. Indeed, binding of these drugs to LTCCs is state dependent, favoring open^8,9^ (e.g., verapamil and diltiazem) and/or inactivated^8,10,11^ (e.g., dihydropyridines, or DHPs) states, conditions that are more readily encountered in cardiovascular tissues than in the brain. Thus, current LTCC drugs may be functionally biased for cardiovascular indications, limiting their therapeutic utility for CNS indications.

VGCCs in vivo are large macromolecular signaling complexes consisting of a central pore-forming α1 subunit surrounded by multiple auxiliary subunits (e.g., β and α2δ) and other interacting/modulatory proteins. A recent study in cardiomyocytes showed that a large variety of proteins lie within the Ca_V_1.2 channel subdomain^12^. A study of Ca_V_2 channel nano-environments similarly revealed that Ca_V_2 channels are embedded into protein networks that may be assembled from a pool of a large number of proteins^13^. A number of studies have characterized regulatory proteins that biochemically and/or functionally interact with neuronal LTCCs^14–22^. In addition, various spliced variants of Ca_V_1.2 have also been identified^23^, some of which may be more neuronally enriched and confer distinct physiological and pharmacological properties^5,24^. Variant isoforms of LTCC genes can lead to changes in channel properties^24–26^. Therefore, it is conceivable that LTCCs in neurons may confer novel and/or neuro-selective pharmacological properties through neuro-favoring/specific interactions.

In this study, we took a phenotypic approach to screening for novel, neuro-selective LTCC blockers. Using primary cultures of rat cortical neurons, we developed two phenotypic assays, a fluorescence-based medium-throughput screening assay and a secondary patch clamp confirmation assay. We conducted a primary screen of 1278 commercially available small molecule drugs and other bioactives, which successfully identified majority of the LTCC drugs, lending validation to these assays. Importantly, we identified a novel, potent LTCC blocker, suggesting the utility of this approach towards identifying novel and neuro-selective LTCC modulators.

## Results

### Isolation of LTCC-mediated responses in rat primary cortical cultures

**Figure 1:**
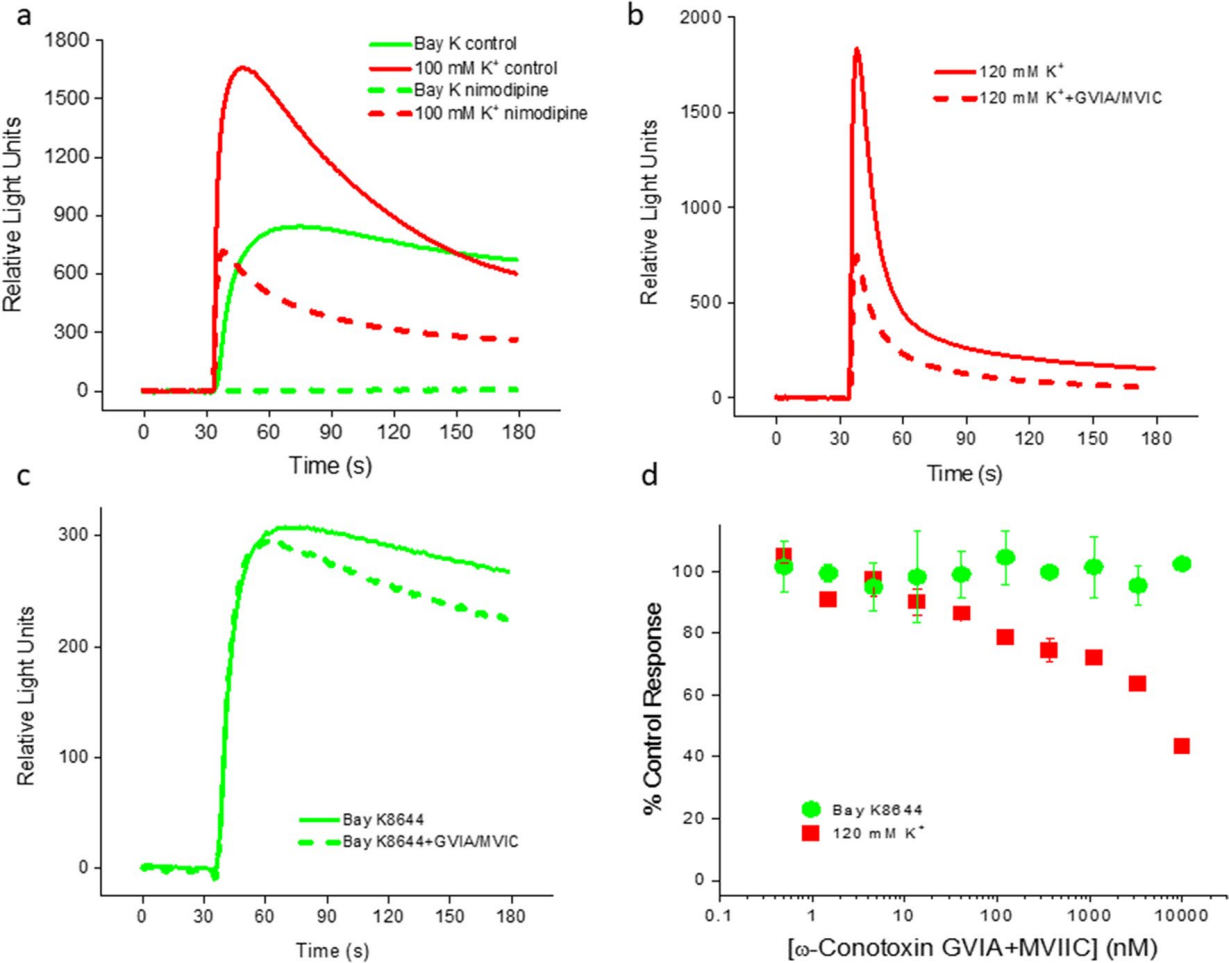
Functional expression of LTCCs and other VGCCs in rat cortical cultures. (**a**) Calcium fluorescent responses to stimulation by high [K^+^] (100 mM; solid red line) and Bay K8644 (1.1 µM; solid green line). Nimodipine (3 µM) partially (dashed red line) and completely (dashed green line) inhibited the high [K^+^]- and Bay K-evoked responses, respectively. (**b**) Calcium fluorescent responses evoked by high [K^+^] (120 mM; solid line) were partially blocked by a cocktail of VGCC (but not LTCC) blockers (dotted line), ω-conotoxin GVIA (N-type) and ω-conotoxin MVIIC (N-, P- and Q-types), both at 10 µM. (**c**) Calcium fluorescent responses evoked by Bay K8644 (300 nM; solid line) were not sensitive to the cocktail (dotted line). (**d**) Concentration dependence of the cocktail effects on high [K^+^]- and Bay K-induced responses. Summary of 4 independent experiments like those in (**b**) and (**c**). The holding [K^+^] was 20 mM in all experiments.

Cortical neurons express LTCCs as well as other VGCCs. As shown in Fig. 1a, application of 100 mM K^+^ evoked a large calcium fluorescent response in a rat primary cortical culture that was partially blocked by nimodipine (3 µM), a potent and selective LTCC antagonist, indicating that LTCCs represented a substantial fraction of VGCCs expressed in these cultures. Elevated concentrations of K^+^ indiscriminately activate all VGCCs. To selectively activate LTCCs, we used Bay K8644, a LTCC-selective agonist, to stimulate responses in these cultures. In contrast to 100 mM K^+^, the Bay K8644 (1.1 µM)-induced response was completely blocked by 3 µM nimodipine (Fig. 1a), indicating that it was LTCC mediated. To better understand whether the Bay K-induced response was a result of direct calcium influx through LTCC channels or due to secondary activation of other VGCCs, we included two VGCC- (but not LTCC-) blocking peptides, ω-conotoxin GVIA (N-type calcium channel blocker) and ω-conotoxin MVIIC (N- and P/Q-type calcium channel blocker). As shown in Fig. 1b–d, the peptide cocktail partially blocked the high [K^+^]-induced response in a concentration-dependent manner, but had little effect on Bay K-induced response, indicating that the Bay K-induced response was due to direct calcium influx through LTCCs.

### Optimization of the phenotypic fluorescence assay

**Figure 2:**
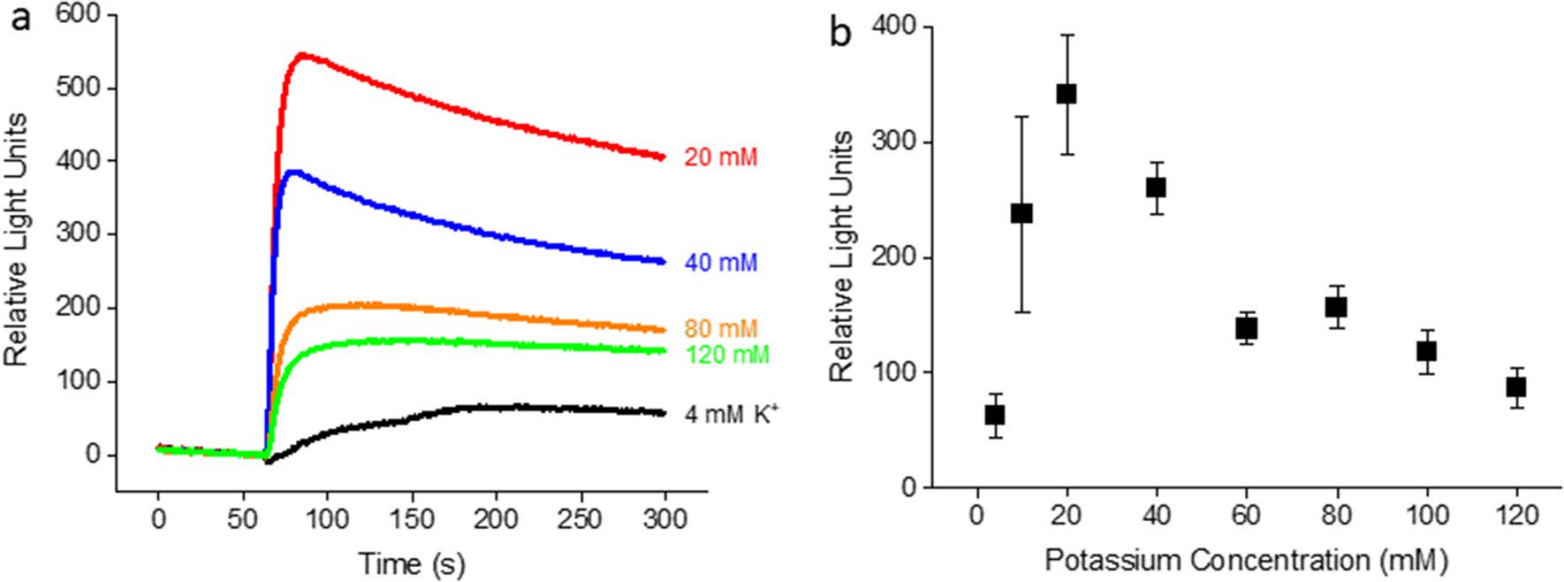
[K^+^] dependence of fluorescent responses to Bay K8644 (300 nM) in rat cortical cultures. (**a**) Traces of calcium fluorescence for a range of K^+^ concentrations as indicated. Each [K^+^] used during Bay K application was the same as that used for preincubation of cells. (**b**) Summary of experiments (n = 10) similar to (**a**).

Bay K8644 potentiates LTCCs by shifting the voltage dependence of channel activation towards hyperpolarized membrane potentials^27^. Consequently, the amplitude of Bay K-evoked response is dependent on the resting membrane potential. Significant responses appear only when the resting membrane potential is depolarized sufficiently close to the threshold for LTCC activation. To maximize Bay K-evoked responses, we varied the resting membrane potential by preincubating cells with a range of holding K^+^ concentrations. As shown in Fig. 2, Bay K (300 nM)-induced responses as a function of holding [K^+^]s were bell-shaped, peaking at 20 mM holding [K^+^], which corresponds to a resting membrane potential of roughly − 50 mV. At this membrane potential, T-type calcium channels are inactivated, preventing potential contamination by non-specific responses from this class of VGCCs. Together with the finding that the Bay K-induced responses at 20 mM holding [K^+^] were LTCC specific (Fig. 1), we chose to preincubate cells in a buffer containing 20 mM K^+^ in our assay.

We also studied the effects of several other parameters on Bay K-evoked responses, including cell density, tolerance for DMSO and culture days in vitro (DIV). The results are either shown in Supplementary Fig. S1 and Fig. S2 or described in Methods section. The values for these parameters used in the screening assay are given in the Methods section.

### Pharmacology of reference compounds

**Figure 3:**
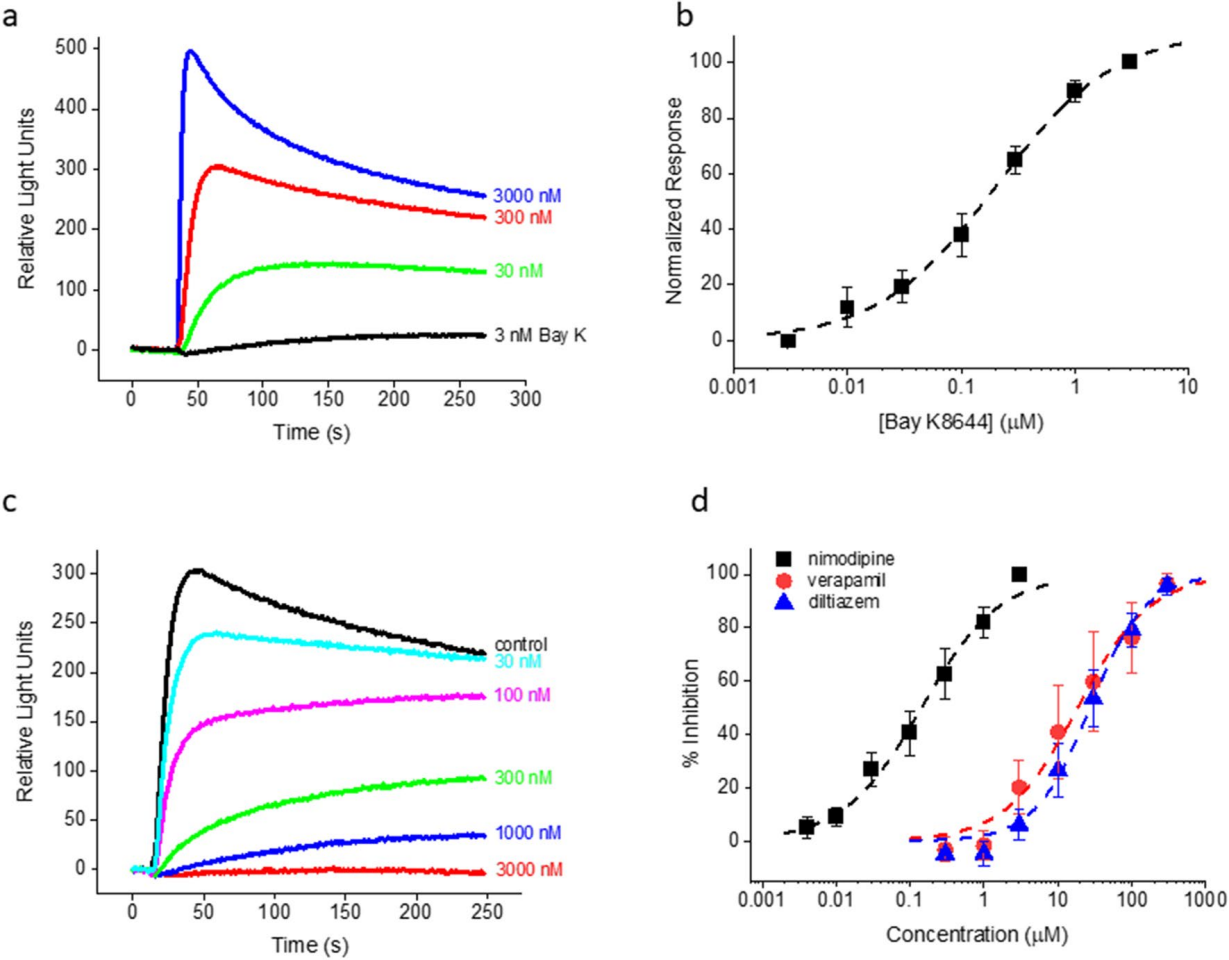
LTCC pharmacology from rat cortical cultures. (**a**) Fluorescent responses to a range of Bay K8644 concentrations. (**b**) Summary of Bay K concentration dependence for seven independent experiments similar to (**a**). EC_50_ = 203.9 nM from the best fit (dashed curve) to the data. (**c**) Fluorescent responses to Bay K8644 for a range of nimodipine concentrations. (**d**) Summary of concentration dependence data for multiple experiments for nimodipine, verapamil and diltiazem. The EC_50_ values from the best fits (dashed curves) to the nimodipine (n = 12), verapamil (n = 4) and diltiazem (n = 6) data were 142.4 nM, 18.5 µM and 27.1 µM, respectively. The concentration of Bay K8644 was 300 nM in (**c**) and (**d**). The holding [K^+^] was 20 mM in all the experiments.

Next, we evaluated the LTCC pharmacology in the calcium flux assay using reference compounds that are well described in the literature. Bay K potently activated LTCCs with an EC_50_ of 203.9 nM (Fig. 3a,b). The three major classes of LTCC antagonists, nimodipine (a DHP), verapamil (a phenylalkylamine) and diltiazem (a benzothiazepine) concentration-dependently inhibited 300 nM Bay K-induced responses with IC_50_ values of 142.4 nM, 18.5 µM and 27.1 µM, respectively (Fig. 3c,d), in general agreement with the literature^28–30^. Taken together, our phenotypic calcium flux assay showed convincing hallmarks of LTCC specificity.

### Prestwick library screen

**Figure 4:**
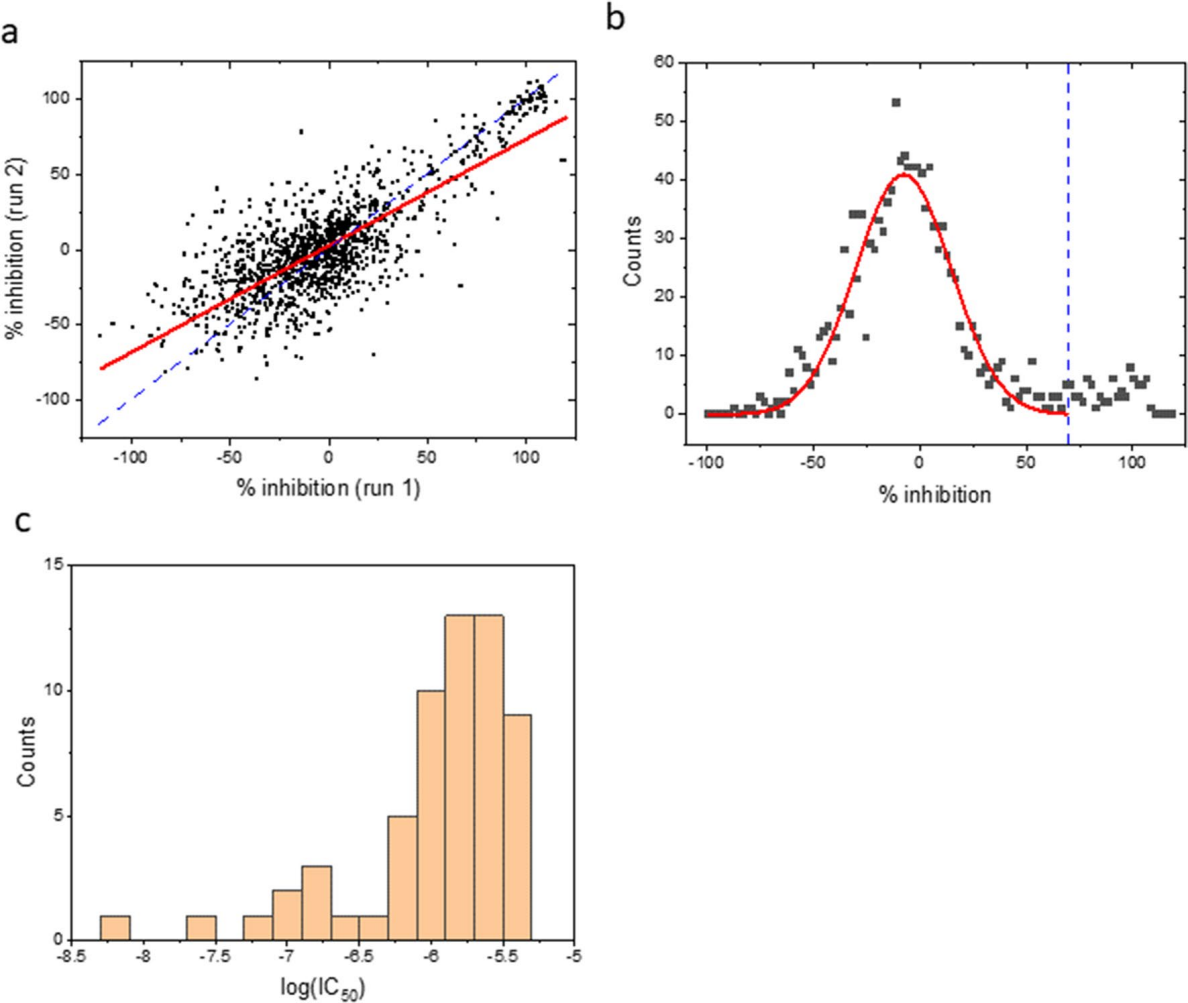
Results of the Prestwick library screen. (**a**) Correlation of two independent runs for each compound (test concentration = 3 µM). Two compounds with large fluorescent artifacts are omitted. The solid red line is the linear least-squares fit (r^2^ = 0.62; slope = 0.71). The dashed blue line is the line of identity. (**b**) Histogram of the mean % inhibition. The bin size is 2%. Data with % inhibition < 70% (left of the dashed blue line) are fitted to a Gaussian function (solid curve; mean ± SD = -7.8 ± 22.5%). (**c**) Potency histogram of confirmed hits from the PW screen. The bin size is 0.2.

To further validate and test the robustness of the assay, we performed a pilot screen of 1278 known bioactive small molecules from the Prestwick (PW) library. Compounds were screened at a single concentration (3 µM) in duplicates and percent inhibition values were calculated for each compound. The Z’ value for the screen was 0.52 ± 0.11 (mean ± SD). The correlation between two independent runs was 0.62 with a slope of 0.71 (Fig. 4a), suggesting reasonable reproducibility. The histogram of the mean % inhibition showed a major peak centered near zero (mean ± SD = -7.8 ± 22.5%) and a second, smaller population with greater % inhibition values (Fig. 4b). We set the cutoff value at 70% based on these data, resulting in the selection of 75 candidate hits (5.9% of the compounds screened) for confirmation in a concentration–response format. Sixty of them (80%) were confirmed with IC_50_ values < 5 µM (n = 3). The profile of the confirmed hits is summarized Fig. 4c.

DHPs are a major class of LTCC-blocking drugs and are well represented in the PW library (12 DHPs). Ten of the 12 DHPs (83.3%) were successfully identified in the top 75 hits from the screen, all of which were among the top 20 confirmed hits with highest potencies. At least six of the remaining 10 non-DHPs in the top 20 hits, cyproheptadine^31^ (IC_50_ = 138 nM from the screen), GBR 12909^32^ (680 nM), lidoflazine^33^ (710 nM), fluspirilene^34^ (760 nM), fendiline^35^ (820 nM), and clomipramine^36^ (1010 nM), have been reported in the literature to potently block LTCCs. In the top 10 confirmed hits listed in Table 1, eight are DHPs and two (niclosamide and cyproheptadine) are non-DHPs. To our knowledge, niclosamide (an anthelmintic; 80 nM) is the only compound in Table 1 for which no published information is available about the effect on LTCCs. Using a separately validated patch clamp assay, we confirmed the LTCC-blocking activity of niclosamide in rat cortical neurons (see below). In conclusion, the phenotypic calcium flux assay was robust and validated by its ability to identify most of the known LTCC blockers as well as a novel LTCC blocker in the PW library.

**Table 1:** Top 10 primary hits from the PW library by potency.

Drug Name	FLIPR IC50 (nM)	Chemical Class	Rat cortex binding Ki (nM)			References (LTCC block)
	Rat cortical culture		DHP site	Phenylalkylamine site	Benzothiazepine site	
Isradipine	8	DHP				Balasubramanian et al., 2009
Nilvadipine	28	DHP				Ishibashi et al., 1997
Felodipine	57	DHP				Angus et al., 2000
Niclosamide	80	Non-DHP	> 30,000	> 30,000	> 30,000	
Benidipine	104	DHP				Nishiya et al., 2002
Nimodipine	110	DHP				Xia et al., 2004
Cyproheptadine	138	Non-DHP	3562	3303	2278	Winquist et al., 1984
Nitrendipine	140	DHP				Xia et al., 2004
Nicardipine	290	DHP				Xia et al., 2004
Lacidipine	453	DHP				Xia et al., 2004

IC_50_ values (nM) are from the phenotypic calcium flux assay using rat cortical cultures. K_i_ values (nM) are from binding studies using rat cortical homogenates.

### Development of a secondary patch clamp assay

**Figure 5:**
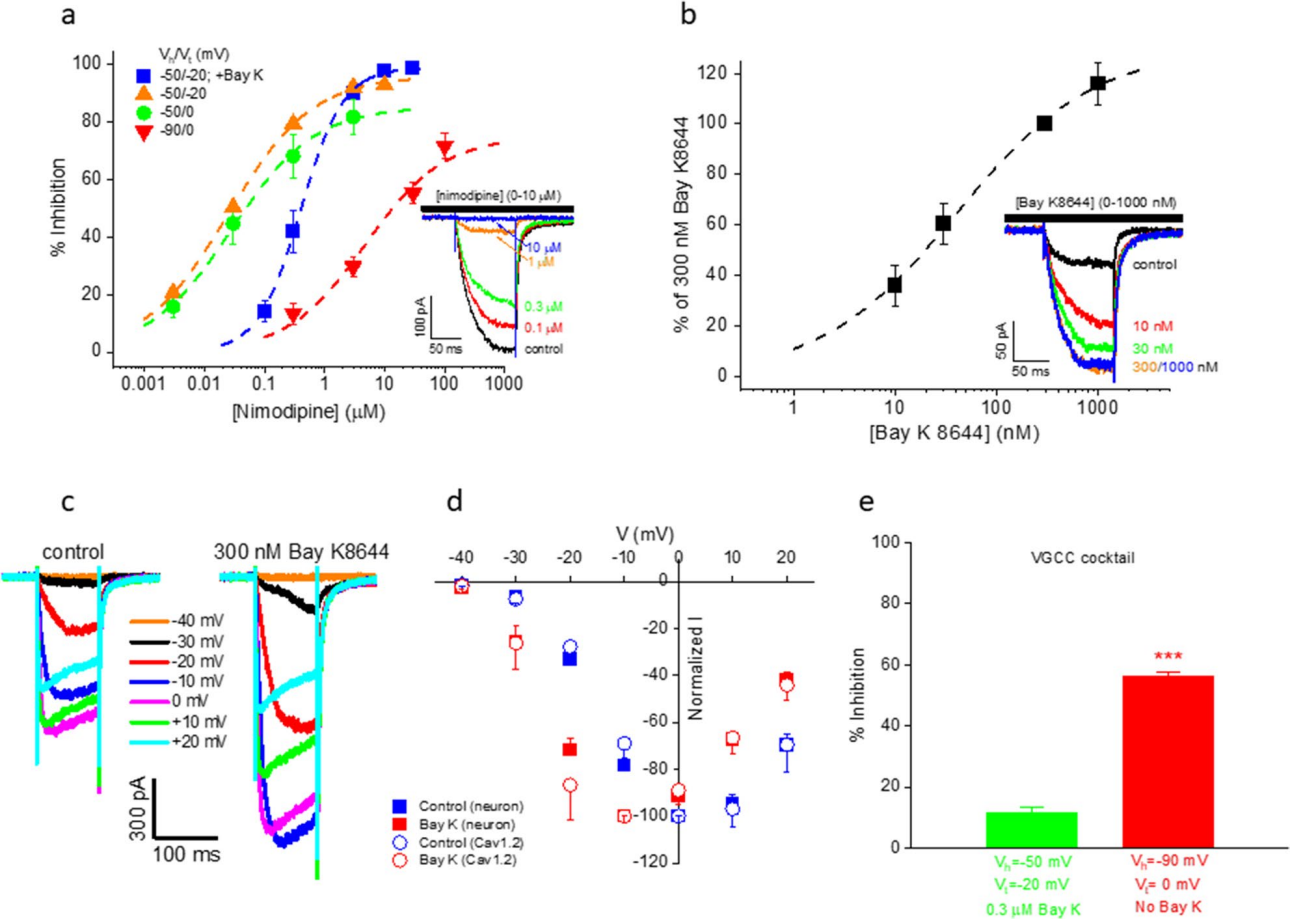
Patch clamp recordings from rat cortical neurons. (**a**) Nimodipine concentration dependence in the absence and presence of 300 nM Bay K8644. V_hold_ (V_h_) and V_test_ (V_t_) are as indicated. IC_50_/max %inhibition from the best fits (dashed lines) are: 28.3 ± 6.5 nM/85.4 ± 3.7% (− 50/0 mV, control, n = 4), 24.8 ± 1.3 nM/96.1 ± 0.8%, (− 50/− 20 mV, control, n = 4), 400.7 ± 21.8 nM/99.1 ± 1.1% (-50/-20 mV, 300 nM Bay K, n = 7), and 4.8 ± 1.4 µM/75% (− 90/0 mV, control, n = 4), respectively. Inset: Current traces for -50/-20 mV with 300 nM Bay K. (b) Bay K8644 concentration dependence. EC_50_ = 40.6 nM from the best fit (dashed line; n = 7). V_hold_ = − 50 mV and V_test_ = -20 mV. Inset: Current traces. (**c**) Current traces from the same neuron in response to a series of step voltage changes (from − 40 mV to + 20 mV) in the absence (left panel) and presence (right panel) of 300 nM Bay K8644. The holding potential was − 50 mV. (**d**) Normalized I-V plots from multiple neurons (solid symbols) and CHOs expressing Ca_V_1.2 (open symbols) in the absence (blue symbols; normalized to the peak current amplitude at 0 mV for each cell before averaging; n = 27 and 8, respectively) and presence (red symbols; normalized to the peak current amplitude at − 10 mV for each cell before averaging; n = 17 and 6, respectively) of 300 nM Bay K8644. The holding potential was − 50 mV for both neurons and CHOs. (**e**) A cocktail of peptidic blockers of VGCCs (but not of LTCCs) (200 nM ω-agatoxin IVA for P-type, 1 µM ω-conotoxin GVIA for N-type, 1 µM ω-conotoxin MVIIC for N-, P- and Q-types and 30 nM SNX-482 for R-type, respectively) had minimal effect on Ba^2+^ currents recorded under conditions favoring LTCC activation (11.3 ± 2.1% inhibition, n = 3; left bar), but blocked a significantly higher fraction of the total current under conditions activating all VGCCs (56.1 ± 1.7% inhibition, n = 6; right bar; p < 0.001, two-tailed Student’s t-test).

We also developed and validated a secondary patch clamp assay using rat cortical neurons to confirm hits identified from the calcium flux assay. The buffers used in patch clamp were intended for isolating/maximizing calcium channel responses (substituting extracellular Ba^2+^ for Ca^2+^) and minimizing/eliminating sodium and potassium channel activity (extracellular choline replacing Na^+^ and intracellular Cs^+^ replacing K^+^). They simultaneously served to abolish network activities normally present in neuronal cultures without the need to introduce cocktail inhibitors of synaptic activity. To obtain conditions optimized for LTCC currents, we varied the holding (− 90 mV or − 50 mV) and testing (− 20 mV or 0 mV) potentials. As shown in Fig. 5a, the fraction of nimodipine-sensitive current was lower when holding/testing the cell at − 90/0 mV (75%) or at − 50/0 mV (85%) compared to the − 50/− 20 mV protocol (96%). Importantly, inclusion of 300 nM Bay K (a near-saturating concentration; Fig. 5b) in the extracellular solution greatly amplified the LTCC currents (Fig. 5c,d) and effectively reduced the fraction of nimodipine-insensitive (i.e., non-LTCC) currents (as seen in Fig. 5a from the 99% block by nimodipine in the presence of Bay K). As expected, Bay K decreased the potency of nimodipine inhibition by 16.7 fold (IC_50_ was increased from 24.8 nM in the absence of Bay K to 414.6 nM in the presence of Bay K; Fig. 5a), consistent with the two DHPs competing for the same binding site^37^. In addition, the potency of nimodipine block was also decreased at the holding potential of − 90 mV (IC_50_ = 4.8 µM; Fig. 5a), in agreement with the well-documented voltage dependence of DHP blockers^8^.

Under the optimized recording conditions (i.e., with the -50/-20 mV protocol in the presence of Bay K), a cocktail of VGCC- (but not LTCC-) blocking peptides had little effect (11% inhibition) on Ba^2+^ currents (Fig. 5e). By contrast, the same cocktail blocked a significantly higher fraction (56%) of Ba^2+^ currents under conditions in which other VGCCs were allowed to contribute to the current (i.e., with the − 90/0 mV protocol in the absence of Bay K; Fig. 5e). Taken together, results in Fig. 5 demonstrated that the chosen recording solutions, inclusion of Bay K and use of the − 50/− 20 mV voltage protocol together enabled the isolation/recording of virtually pure LTCC currents from cortical neurons.

### Confirmation of primary hits in patch clamp

**Figure 6:**
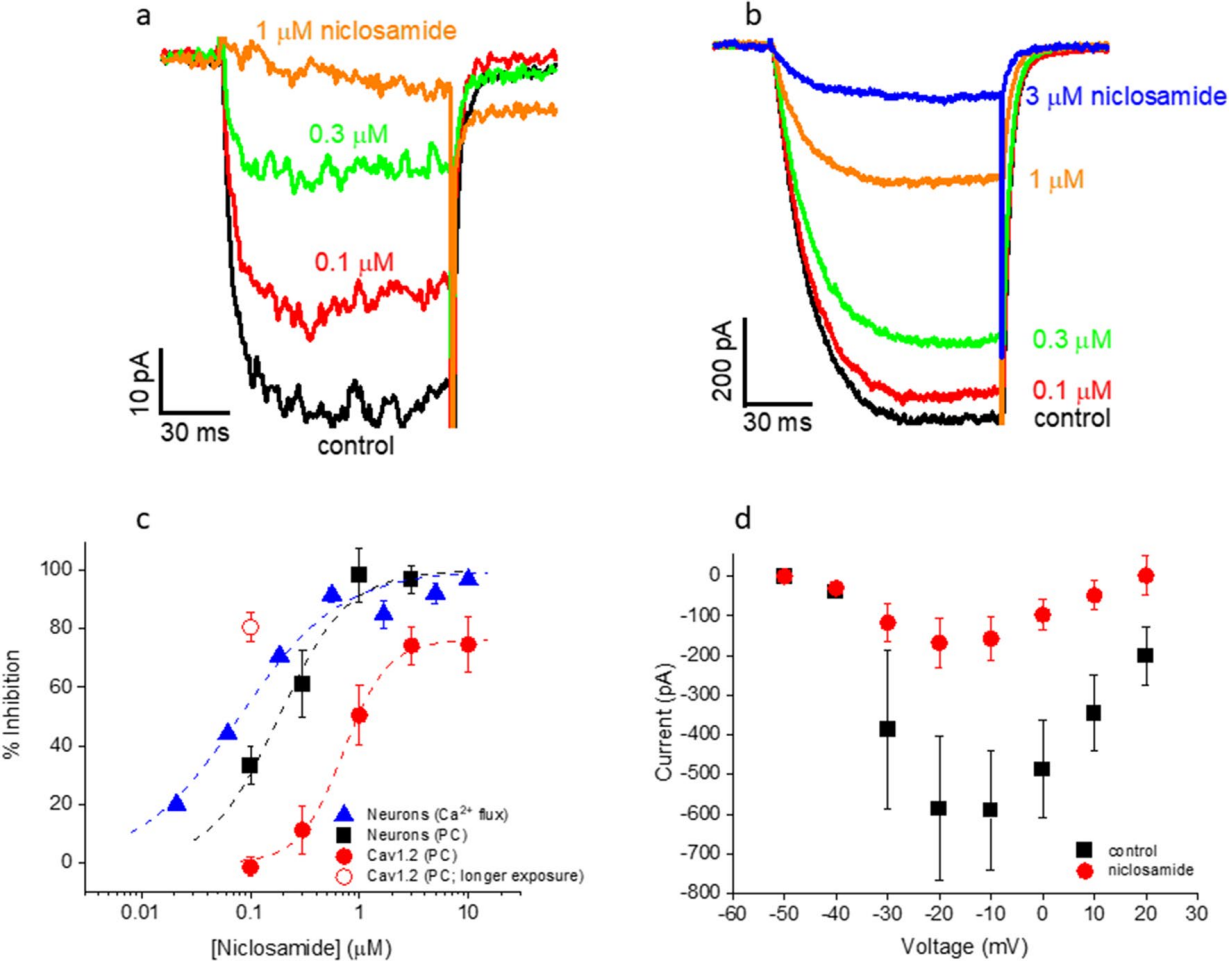
Effect of niclosamide on LTCC currents from rat cortical neurons and Ca_V_1.2 currents in CHO cells . (**a**) Current traces from a neuron in the absence and presence of niclosamide. (**b**) Current traces from a Ca_V_1.2-expressing CHO in the absence and presence of niclosamide. (**c**) Concentration-dependent inhibition of LTCC-mediated calcium flux in cortical cultures (solid triangles; IC_50_ = 79.5 ± 12.8 nM; maximal % inhibition = 100%; n = 3), LTCC currents recorded from cortical neurons (solid squares; IC_50_ = 183.1 ± 28.2 nM; maximal % inhibition = 100%; n = 6) and Ca_V_1.2-expressing CHO cells (solid circles; IC_50_ = 715.3 ± 49.2 nM; maximal % inhibition = 76.1 ± 2.0%; n = 7; p < 0.001 vs neurons, two-way ANOVA). At 100 nM niclosamide, inhibition of Ca_V_1.2 was significantly greater when CHO cells were exposed to the compound for longer periods (open circle, 80.6 ± 4.9%, n = 4) than that of LTCCs in neurons (solid squares; p < 0.001, one-way ANOVA) or that of Ca_V_1.2 when CHO cells were exposed to the compound for shorter periods (solid circles; p < 0.001, one-way ANOVA). All patch clamp experiments were performed in the presence of 300 nM Bay K8644 at a holding potential of -50 mV and test potential of − 20 mV. (**d**) I-V plots in the absence (squares) and presence (circles) of 200 nM niclosamide (from the same 5 neurons). The holding potential was − 50 mV. A cocktail of VGCC blockers, SNX-482 (30 nM), ɷ-agatoxin IVA (200 nM), ɷ-conotoxin GVIA (1 µM) and ɷ-conotoxin MVIIC (1 µM), was present in the extracellular solution.

Using the patch clamp assay validated above, we tested niclosamide and cyproheptadine, the two non-DHPs hits in Table 1. In rat cortical neurons, 3 µM cyproheptadine blocked 75.2 ± 9.1% (n = 4) of the LTCC currents, in line with its binding affinities to the DHP, phenylalkylamine and benzothiazepine sites (Table 1). In contrast, while niclosamide potently blocked neuronal LTCC currents (IC_50_ = 183.1 ± 28.2 nM, n = 6; Fig. 6a,c), it showed no binding at any of these sites in rat cortical preparations (K_i_ > 30 µM for all three sites; n = 4; also see Table 1), suggesting that niclosamide binds to a novel site in the LTCC channel complex in neurons. Similar to control, the I-V curve in the presence of niclosamide (200 nM) peaked near − 20 mV, although niclosamide block was more pronounced at depolarized potentials (Fig. 6d). The activation and inactivation kinetics in the presence of niclosamide were not significantly different from control. The 20–80% rise time (at − 20 mV) was 17.4 ± 2.9 ms (n = 5) and 16.1 ± 3.5 ms (n = 4) in the absence and presence of 200 nM niclosamide, respectively (p > 0.7). The current remaining after 400 ms of depolarization to − 20 mV was 41.9 ± 7.5% (n = 4) of the peak in niclosamide vs 49.1 ± 3.4% (n = 5) in control (p > 0.3). Both compounds also blocked Ca_V_1.2 currents in CHO cells (IC_50_ = 715.3 ± 49.2 nM for niclosamide, n = 7, Fig. 6b,c; 52.3 ± 6.1% inhibition at 3 µM for cyproheptadine, n = 3). Niclosamide blocked Ca_V_1.2 expressed in CHO cells with lower potency (by ~ fourfold) and efficacy (76.1%) than for neuronal LTCCs (full efficacy). Surprisingly, niclosamide block of Cav1.2 (100 nM) was more pronounced in experiments in which niclosamide was applied for significantly longer durations (average of ~ 6.5 min) than those used for Ca_V_1.2 concentration–response experiments (average ~ 3.5 min) (Fig. 6c). Though not corrected for current rundown, this extra block was not due to compound-independent current rundown, which was only 8.4 ± 3.7% and 12.4 ± 7.6%, respectively, after 3.5 and 6.5 min in compound-free buffer (n = 4).

## Discussion

In an effort to identify novel, neuro-selective LTCC blockers, we took a phenotypic approach and developed two assays using rat primary cortical cultures: a medium-throughput, calcium flux-based fluorescence assay and a patch clamp, secondary confirmation assay.

We optimized the assays by using Bay K8644, a LTCC-selective agonist, along with a moderately elevated [K^+^] in the extracellular incubating buffer (20 mM; the calcium flux assay) or moderately depolarized holding potential (− 50 mV; the patch clamp assay). This combination selectively amplified LTCC responses with minimal activation of other VGCCs. We further showed that these protocols resulted in Ca^2+^ responses or Ba^2+^ currents specifically through LTCCs rather than non-specific or LTCC-mediated secondary responses. Moreover, the pharmacology of LTCCs using well-documented reference compounds was also in line with the literature.

Brain LTCCs comprise Ca_V_1.2 and Ca_V_1.3, with the former being the dominant isoform^38^. While it is tempting to speculate that responses in our assays primarily reflect Ca^2+^/Ba^2+^ influx through Ca_V_1.2, it is not possible to estimate the relative contributions of these isoforms based on our data.

We conducted a pilot screen of 1278 known bioactive agents from the PW library using the calcium flux assay. The results of the screen validated our assay in several ways. First, the assay was robust as suggested by (1) a good correlation (r^2^ = 0.62) between the two independent runs of the screen, (2) a hit confirmation rate of 80% in a concentration-dependent manner, and (3) a Z’ value of ~ 0.5. Second, most of the DHP drugs in the library (~ 83%), which are all potent LTCC blockers, were correctly identified in the screen. They were all among and represented 50% of the top 20 most potent hits. Third, six of the 10 non-DHPs in the top 20 hits have been reported as LTCC blockers in the literature. The only two non-DHPs in the top 10 hits were both confirmed in the patch clamp assay. One of them, niclosamide, is a novel LTCC blocker identified from our screen for the first time. Independent confirmation of these compounds by patch clamp lent further support for the strength and validity of our primary screening assay.

Combining the literature information and our patch clamp results for the top 20 most potent hits, an upper limit can be placed for the false positive rate associated with the screen. Since no literature information on LTCC effects is available for 3 of the 20 hits (the 4th, niclosamide, was positively confirmed by our patch clamp assay), the false positive rate could be up to 15% (3/20) assuming all three are false positives. It should be cautioned, however, that this estimate could be less reliable when extrapolated to a larger-scale screen because of (1) the relatively small data set in this study and (2) the nature of the extrapolation, i.e., largely from one known mechanism, DHP block of LTCCs, to potentially different and/or novel mechanisms by which many hits cause apparent inhibition.

It should be noted that it is intrinsically a higher hurdle to demonstrate that a hit in our primary assay (or any phenotypic screen) is a false positive since negative outcomes (i.e., failure to confirm a hit) in confirmation assays using recombinant Ca_V_1.2-expressing systems do not necessarily prove that a hit is a false positive. After all, our phenotypic assay is intended for identifying neuro-selective or otherwise mechanistically novel LTCC antagonists, which may well be inactive at recombinant Ca_V_1.2-expressing cells if, for instance, the target protein that a hit binds to is not expressed in the recombinant system. Nor is lack of displacement binding, even in the same neuronal preps, indicative of false positivity as the compound may bind to a different site than the tracer used (as illustrated for niclosamide in this study). Therefore, it is critical to verify primary hits from a phenotypic screen by an independent, preferably functional assay (e.g., patch clamp) in a native environment.

Using cortical neurons prepared the same way as for the calcium flux assay, we developed and validated a manual patch clamp assay for hit confirmation. The patch clamp assay confirmed both non-DHPs in the top 10 primary hits, niclosamide and cyproheptadine. Of particular interest, niclosamide has not been noted in the literature for its actions at LTCCs. Combining patch clamp with binding studies, we showed that niclosamide bound to a novel site in neuronal LTCCs with at least two orders of magnitude higher affinity than that at the DHP, phenylalkylamine and benzothiazepine sites. Future studies will be necessary to characterize the novel binding site and molecular mechanism of niclosamide.

In summary, we have developed and validated two phenotypic assays that have promising potential for identifying mechanistically novel and neuro-selective LTCC blockers in large-scale screening campaigns.

## Methods

### Neuronal cultures

Rat cortical neurons were isolated from E-18 Sprague–Dawley rats as previously described^39^ (BrainBits, Springfield, IL). Briefly, cortices were isolated, rapidly removed from decapitated pups, and stored in ice-cold Hibernate-E media. Cortices were then transferred into a 15 mL conical tube and incubated at 37 °C for 10 min in 2 mL papain dilution buffer containing 5 mM L-cysteine, 1 mM EDTA, 10 mM HEPES, 100 µg/mL BSA, 1% papain and 0.1% DNase I. Heat-inactivated FBS (2 mL) and additional DNase I (5 µL) were added at the end of the incubation period after which cells were mechanically dissociated until homogeneous using a 10 mL pipette and centrifuged (5 min at 1000 rpm at room temperature). The cell pellet was then resuspended in culture media containing neurobasal medium supplemented with B27 and 0.5 mM Glutamax. Resuspended cells were centrifuged and the pellet was resuspended again in culture media. Finally, cells were filtered using a 70 µm cell strainer, centrifuged, resuspended in culture media, and plated in Falcon 384-well black wall, clear bottom plates (for the calcium flux assay) or on poly-D-Lysine coated glass coverslips (for the patch clamp assay). Cortical cultures were placed in a 37 °C humidified incubator maintained at 5% CO_2_. One half of the culture media was replaced with fresh media 5 days after plating. Animal protocols were approved by the Institutional Animal Care and Use Committee of Janssen Research & Development, L.L.C. All experiments were performed in accordance with the relevant guidelines and regulations.

### Recombinant cell cultures

The tetracycline-inducible human Ca_V_1.2 α1 subunit was stably co-expressed in CHO cells with human β2a and human α2δ1 subunits (with GenBank accession numbers NM_000719.4, NM_000724.2, and NM_000722.2, respectively; Charles River, Cleveland, OH). The CHO cells also stably expressed hKir2.2. Cells were cultured in Ham’s F12, supplemented with 10% FBS, 1% penicillin–streptomycin, 0.01 mg/mL blasticidin, 0.25 mg/mL G418, 0.25 mg/mL hygromycin, and 0.4 mg/mL zeocin. and incubated at 37 °C with 5% CO_2_. Cells were induced with 1 µg/mL tetracycline in selection antibiotic-free media the day before use.

### Calcium flux assay

The assay buffer contained 137 mM NaCl, 20 mM KCl, 10 mM HEPES, 5 mM glucose, 2 mM CaCl_2_, 1 mM MgCl_2_, at pH 7.4. For experiments in which the KCl concentration was varied, the concentration of NaCl was adjusted accordingly to maintain a constant total salt concentration. The cocktail used to inhibit the network activity contained 0.1 µM tetrodotoxin (TTX), 10 µM picrotoxin, 50 µM D-(-)-2-Amino-5-phosphonopentanoic acid (AP5) and 5 µM 2,3-Dioxo-6-nitro-1,2,3,4-tetrahydrobenzo[f]quinoxaline-7-sulfonamide (NBQX).

Assay development experiments were conducted using DIV11-21 primary cultures at a density of 12–24 K/well, for which Bay K-induced responses did not vary widely. The PW library screen was performed using DIV12 cultures, with a density of 12 K/well. Bay K8644 (1.1 µM) and nimodipine (3 µM) were used as positive and negative controls, respectively. Cells were first washed 3 × with 85 µL assay buffer using the ELX-405 plate washer, ending with 25 µL buffer in each well. Then, 25 µL of 1 × Calcium 6 no-wash dye was added to each well using a Multidrop and incubated at room temperature for 1 h. Next, 25 µL of 6 × compound prepared in cocktail assay buffer was added and incubated for 30 min at room temperature. The compound concentration during incubation was 2 × . The plates were aspirated, leaving 25 µL/well. Finally, assay plates were transferred to FLIPR (Molecular Devices, Sunnyvale, CA) for a 30 s baseline read followed by addition of 2 × Bay K 8644 (2.2 µM). The total recording time was 180 s at 1 Hz. Control wells contained either 1.1 µM Bay K8644 or 1.1 µM Bay K8644 plus 3 µM nimodipine. For the PW library screen, all wells contained either 0.15% (3 µM single concentration screen) or 0.3% (concentration–response confirmation of hits) DMSO. In experiments involving peptidic blockers of VGCCs, 0.1% BSA was included in all buffers.

### Electrophysiology

Patch clamp recordings were conducted at room temperature (~ 22 °C). Whole-cell currents were measured using pClamp 10 software (Molecular Devices) and Axopatch 200B amplifier, lowpass filtered at 2 kHz and digitized at 10 kHz. The series resistance was 75% compensated. In the optimized assay, currents were elicited by depolarization to -20 mV (100 ms) from a holding potential of -50 mV once every 20 s in the constant presence of 300 nM Bay K8644 and allowed to stabilize in both control buffer and compound applications (for ~ 3 min). Compounds were perfused using a SF-77B Fast-Step Perfusion device (Warner Instruments).

DIV5-14 neurons on 35 mm glass coverslips (1–3 × 10^5^/coverslip) or freshly dissociated and tetracycline-induced (for 24–48 h) CHO cells co-expressing human L-type calcium channel α_1C_ (Ca_V_1.2), β_2a_, and α_2_δ_1_ subunits were placed in a chamber on the stage of an inverted microscope and perfused (∼1 mL/min) with an extracellular solution containing: 149 mM choline chloride, 5 mM BaCl_2_, 1 mM MgCl_2_, 5 mM glucose, 10 mM HEPES, at pH 7.4 and 310 mosM/L. Pipette electrodes were filled with an intracellular solution of the following composition: 115 mM Cs methanesulfonate, 20 mM CsCl, 4 mM MgATP, 0.3 mM Na_2_GTP, 10 mM EGTA and 20 mM HEPES, at pH 7.2 and 295 mosM/L. In experiments involving peptidic blockers of VGCCs, 0.1% BSA was included in all extracellular buffers.

### Binding studies

The in-vitro binding studies were conducted by Eurofins (France) using rat cortex. The radio ligands and incubation (at room temperature) times used for the DHP, benzothiazepine and phenylalkylamine sites were [^3^H]nitrendipine (0.1 nM; 90 min), [^3^H]diltiazem (15 nM; 120 min) and [^3^H]D888 (3 nM; 120 min), respectively. The amount of protein/assay were 150 µg, 300 µg and 200–300 µg, respectively. The ratio of specific over non-specific binding signals was 9.1–15.1, 2.8–3.5 and 5.1–7.8, respectively. Nitrendipine (1 μM), diltiazem (10 μM) and D600 (10 μM), respectively, were used for evaluations of non-specific binding and as positive controls with IC_50_ values of 0.19 ± 0.03 nM, 42.7 ± 6.8 nM and 26.8 ± 3.6 nM, respectively (n = 4).

### Chemicals

TTX was obtained from Alamone (Israel). The PW chemical library was from Prestwick Chemical (France) and contained 1278 small molecular-weight drugs or otherwise bioactive agents. All other reference compounds were from Tocris.

### Data analysis

For the calcium flux assay, the peak Bay K-induced change in fluorescence from baseline, R, was used to calculate the % inhibition value as follows: R = 100*(P-R)/(P-N), where P and N are the peak responses in Bay K alone and Bay K + nimodipine, respectively. Compounds that substantially altered the baseline fluorescence in either direction (≥ ± 30%) were excluded before hit confirmation. For IC_50_ determination, concentration–response data were fitted to a logistic function of the form: R = (A_1_−A_2_)/(1 + (C/IC_50_)^h^) + A_2_, where R was the normalized response, C was the compound concentration, IC_50_ was the concentration at which half-maximal response occurred, h was the Hill coefficient, and A1 and A2 were constants. To evaluate screen robustness, the Z' value was calculated as follows: Z' = 1–(3*SD_positive_ + 3*SD_negative_)/(mean_positive_-mean_negative_), where SD is the standard deviation and positive and negative refer to Bay K alone and Bay K + nimodipine, respectively. Statistical analyses were performed using two-tailed Student’s t-test or one- or two-way ANOVA with post-hoc Tukey test as indicated.

The percent inhibition data from the PW screen were fitted to a Gaussian function of the form: y = y_0_ + A/(w*sqrt(π))*exp(-2*(x-x_c_)^2^/w^2^), with fitting parameters y_0_ as the offset (fixed to zero), A as the area under the curve, x_c_ as center of the peak, and w (= 2.35 SD) as the full width at half maximum (FWHM).

For patch clamp, leak currents were subtracted using a p/4 protocol. For I-V curves, test voltage-evoked peak currents were normalized for each cell before averaging. Concentration–response data were fitted to the same logistic function as for the calcium flux data. Data recorded from n different cells were reported as mean ± SEM. Methods of statistical tests were indicated in figure legends. Data fitting and statistical analysis above were performed using Origin v.9 (Originlab, Northampton, MA).

For binding experiments, results were expressed (1) as percent of control specific binding: 100 × (measured specific binding/control specific binding) or (2) as percent inhibition of control specific binding in the presence of the test compounds:100–100 × (measured specific binding/control specific binding). The IC_50_ values (the concentration that causes half-maximal inhibition of control specific binding) were determined by non-linear regression analysis of the competition curves using Hill equation: Y = D + (A−D)/[1 + (C/IC_50_)]^nH^, where Y = specific binding, A = left asymptote of the curve, D = right asymptote of the curve, C = compound concentration, and nH = slope factor. Analyses were performed using software developed at Eurofins (Hill software) and validated by comparison with data generated by the commercial software SigmaPlot 4.0. The inhibition constants (K_i_) were calculated using the Cheng Prusoff equation K_i_ = IC_50_ × (1 + L/K_D_), where L = concentration of radioligand in the assay and K_D_ = affinity of the radioligand for the receptor. K_D_ values were determined from Scatchard plots.

## Supplementary Information

Supplementary information.

## Data Availability

The datasets generated and/or analyzed during the current study are available from the corresponding author on reasonable request.
