# Supplementary material for: Development of phenotypic assays for identifying novel blockers of L-type calcium channels in neurons
Source: Sci Rep. 2021 Jan 11;11:456. doi: 10.1038/s41598-020-80692-5 (PMC7801380; doi:10.1038/s41598-020-80692-5)
Supplement: Supplementary file 1 — Supplementary information. [file 41598_2020_80692_MOESM1_ESM.pdf]

Development of phenotypic assays for identifying novel blockers of L-type calcium channels in neurons

Rebecca Hagan[1], Elizabeth Rex[2], David Woody[2], Monika Milewski[2], Thomas Glaza[2], Michael P. Maher[1] and Yi Liu[1*]

Neuroscience Discovery[1] and Discovery Sciences[2], Janssen Research & Development, L.L.C.,

3210 Merryfield Row, San Diego, CA, 92121, USA

*Corresponding author: Yi Liu

Email: yliu10@its.jnj.com

Tel: 1-858-784-3196

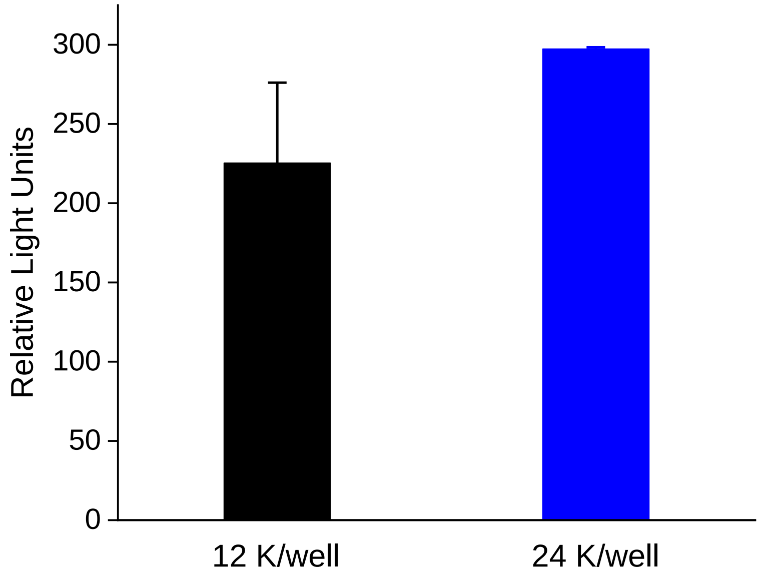

Supplementary Figure 1. Bay K8644 (1.1 μM)-induced fluorescent responses at two cell densities (12 and 24K/well). The responses at the two densities are not significantly different ($p>0.2$; $n=3$ each).

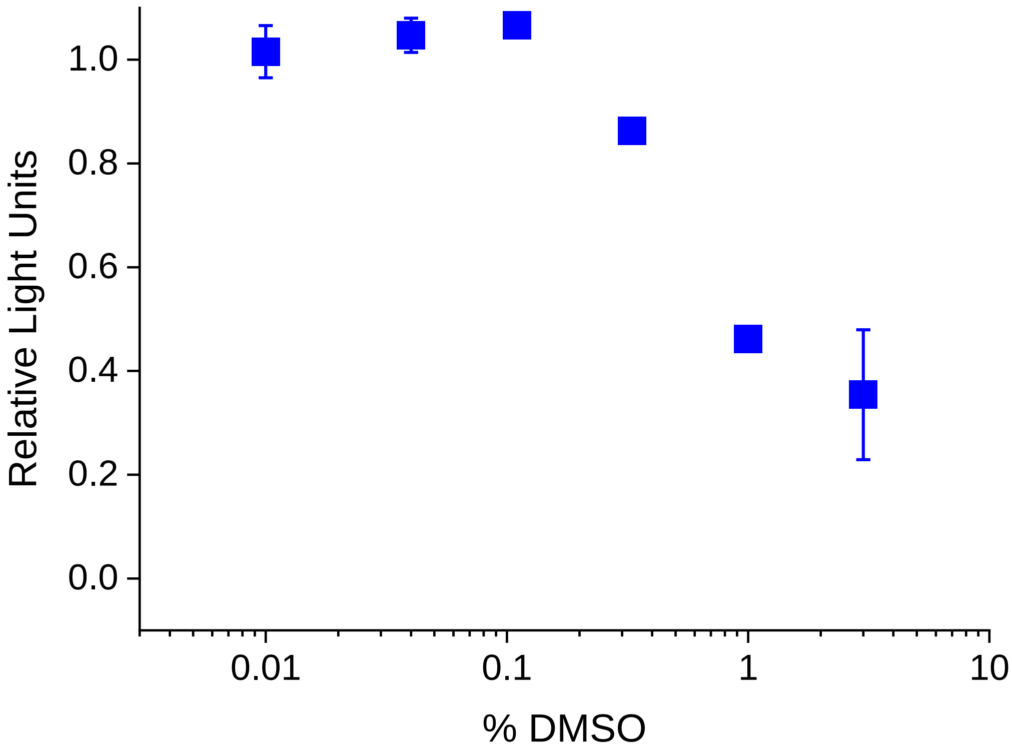

Supplementary Figure 2. DMSO concentration-dependently inhibited Bay K (1.1 µM)-induced fluorescent responses (n=4).
